# Association of Mismatch Repair Mutation With Age at Cancer Onset in Lynch Syndrome: Implications for Stratified Surveillance Strategies

**DOI:** 10.1001/jamaoncol.2017.0619

**Published:** 2017-08-03

**Authors:** Neil A. J. Ryan, Julie Morris, Kate Green, Fiona Lalloo, Emma R. Woodward, James Hill, Emma J. Crosbie, D. Gareth Evans

**Affiliations:** 1 Division of Molecular and Clinical Cancer Sciences, Faculty of Biology, Medicine and Health, University of Manchester, St Mary’s Hospital, Manchester, England; 2 Division of Evolution and Genomic Sciences, University of Manchester, St Mary’s Hospital, Manchester, England; 3 Department of Medical Statistics, University Hospital of South Manchester, Manchester, England; 4 Manchester Centre for Genomic Medicine, Central Manchester University Hospitals NHS Foundation Trust, Manchester Academic Health Science Centre, Manchester, England; 5 Department of Surgery, Manchester Royal Infirmary, Central Manchester University Hospitals NHS Foundation Trust, Manchester Academic Health Science Centre, Manchester, England; 6 Department of Obstetrics and Gynaecology, St Mary’s Hospital, Central Manchester University Hospitals NHS Foundation Trust, Manchester Academic Health Science Centre, Manchester, England

## Abstract

**Importance:**

Lynch syndrome is caused by dominantly inherited germline mutations that predispose individuals to colorectal, endometrial, ovarian, and other cancers through inactivation of the cellular mismatch repair system. Lynch syndrome–associated cancers are amenable to surveillance strategies that may improve survival. The age at which surveillance should start is disputed.

**Objective:**

To determine whether mutated gene and type of mutation influence age at onset of Lynch syndrome–associated cancers.

**Design, Setting, and Participants:**

A retrospective cohort study of individuals with Lynch syndrome–associated colorectal, endometrial, and/or ovarian cancers whose medical records were included in the clinical database of a large quaternary referral center for genomic medicine in the Northwest of England.

**Exposures:**

Mutated gene (*MLH1, MSH2, MSH6*, and/or* PMS2*) and type of mutation (truncating, splicing, or large rearrangement).

**Main Outcomes and Measures:**

Age at cancer diagnosis.

**Results:**

A total of 1063 individuals with proven Lynch syndrome were included, 495 male and 568 female (mean age 52 years; age range, 10-93 years [children were included in the database, but no children developed cancer]). There were 546 men and women with colorectal cancer, 162 women with endometrial cancer, and 49 women with ovarian cancer; mean follow-up was 68.2 months. Among *MLH1* mutation carriers, mutations in *MLH1* were associated with colorectal cancer in 249 (61%) of 409 men and women; endometrial cancer in 53 of 196 (27%) women; and ovarian cancer in 15 (8%) of 196 women. Among *MSH2* mutation carriers, mutations in *MSH2* (the most prevalent mutations overall) were most commonly associated with female-specific cancers: endometrial cancer in 83 (30%) of 279 women; ovarian cancer in 28 (10%) of 279 women; and colorectal cancer in 239 (50%) 479 men and women. Mutations in *MSH6* were less prevalent, and *MSH6* mutation carriers presented with colorectal and endometrial cancer at later ages than carriers of mutations in *MSH2* or *MLH1*. When stratified by mutation type, women with truncating *MLH1* mutations had later ages of onset of endometrial cancer than those with nontruncating mutations (median difference, 6.6 years; 95% CI, 2.7-10.4; *P* = .002). Carriers of truncating *MLH1* mutations presented with colorectal cancer at later ages than those with other mutations, but the difference was not statistically significant.

**Conclusions and Relevance:**

Individuals with known Lynch syndrome could be risk stratified by mutated gene and mutation type in tailored surveillance programs. Specifically, individuals with *MSH6* mutations could be offered cancer surveillance from a later age. Furthermore, those with truncating *MLH1* mutations could begin endometrial cancer surveillance later than those with nontruncating mutations.

## Key Points

**Question:**

Is mutation type associated with the age at onset of Lynch syndrome–associated cancers?

**Findings:**

In this retrospective cohort study of 1063 individuals with proven Lynch syndrome, men and women with *MSH6* mutations were diagnosed with colorectal and endometrial cancer at later ages than those with other mutations. Similarly, women with truncating *MLH1* mutations were diagnosed with endometrial cancer at later ages than those with other mutations.

**Meaning:**

Individuals with known Lynch syndrome could be risk stratified by gene mutation and mutation type in tailored cancer surveillance programs.

## Teaser

In this cohort study of patients with Lynch syndrome with colorectal, endometrial, and/or ovarian cancer, mutation genes and types are evaluated for their association with age at cancer onset.

## Introduction

Lynch syndrome (LS) is caused by germline mutations in one of the mismatch repair genes (*MLH1, MSH2, MSH6*, or *PMS2*) and is associated with increased cancer risk, including colorectal cancer (CRC), endometrial cancer (EC), and ovarian cancer (OC).^1^ Cancer surveillance by colonoscopy has been shown to reduce mortality from CRC.^2^ Gynecological surveillance by transvaginal ultrasonography, hysteroscopy, and endometrial biopsy is of unproven benefit.^4^ The age at initiation of cancer surveillance is also controversial.^6^ The debate is complicated by the rarity of LS and the lack of LS data. Specific gene mutations affect penetrance and influence the age at onset of LS-associated cancers. The aim of this study was to determine whether mutated gene and mutation type affect age at onset of LS-associated cancers.

## Methods

A single-center retrospective cohort study was conducted. The Genetic Register Lynch Syndrome Database from Manchester Centre for Genomic Medicine (large quaternary referral center for 5.6 million people) was interrogated for incident CRC, EC, and OC. All patients provided a priori consent for their data to be used in research; data are anonymized and analyzed as part of clinical audit, and so no ethical review was required. Women were censored from EC and OC analysis at the time of hysterectomy or bilateral salpingo-oophorectomy. All those who did not develop cancer were censored at death or the last recorded date of follow-up.

Gene sequencing of index individuals was performed as previously described^7^ by either Sanger techniques or by next generation sequencing (after 2013) and multiple ligation–dependent probe amplification (MLPA). Only those with heterozygous mutations were included. Statistical analysis was conducted using Stata SE (version 13) and Graphpad Prism (version 7) using Kruskal-Wallis and Mann-Whitney tests and Kaplan-Meier analysis. Significance was defined as *P* ≤ .05. Analysis by gene and mutation type was prespecified as the main comparator for cancer incidence.

## Results

The data set included 1063 individuals (495 men, 568 women) with confirmed pathogenic LS germline mutations. Mean follow-up was 68.2 months.

### Endometrial Cancer

Overall, 162 ECs (eTable in the Supplement) were diagnosed. Of these, 26 ECs were diagnosed among the 68 women with *MSH6* mutations (38%); 83 ECs among the 279 women with *MSH2* mutations (30%); and 53 ECs among the 196 women with *MLH1* mutations (27%). Women with *MSH6* mutations presented with EC at later ages than women with other mutations: median difference from *MLH1*, 3.9 years (95% CI, 0.9-6.5 years); median difference from *MSH2*, 5.7 years (95% CI, 2.4-8.5) (analysis of variance *P* = .002). The median ages of EC onset were 49 (range, 17-71), 47 (range, 32-72) and 53 (range, 42-66) years for women with *MLH1*, *MSH2,* and *MSH6* mutations, respectively. Cumulative incidence of EC according to mutated gene is shown in the Figure. When stratified by mutation type, women with truncating *MLH1* mutations presented with EC at later ages than those with nontruncating mutations (median difference, 6.6 years; 95% CI, 2.7-10.4 years; *P* = .002). The same was not true for *MSH2* carriers and* MSH6* mutation carriers.

**Figure.**
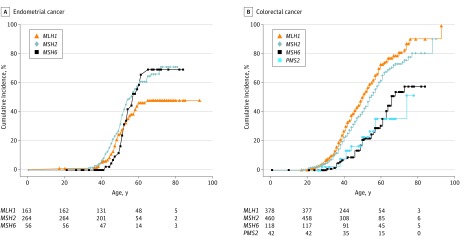
Cumulative Cancer Incidence Stratified by Gene Mutation Later age at onset is seen in both endometrial cancer and colorectal cancer associated with *MSH6* mutation (A and B) and in colorectal cancer associated with *PMS2* mutation (B).

### Ovarian Cancer

Forty-nine OCs were diagnosed, 9 of which were synchronous with, but not metastatic from, EC. The OC rates associated with the evaluated mutated genes were as follows: *MSH2*, 10% (29 of 279); *MLH1*, 8% (15 of 196); and *MSH6*, 7% (5 of 68) (eTable in the Supplement). The median age at OC diagnosis was 47 years (range, 24-70 years); however, women with truncating mutations were older at the time of diagnosis than those with nontruncating mutations (median difference, 6.3 years (95% CI, 0.2-14.2 years; *P* = .04). This was true across all genes but not when individual genes were analyzed separately.

### Colorectal Cancer

Colorectal cancer was diagnosed in 546 individuals (241 women and 305 men) at the following mutated gene–associated rates: *MLH1*, 61% (249 of 409); *MSH2*, 50% (239 of 479); *MSH6*, 33% (43 of 129); and *PMS2*, 32% (15 of 46) eTable in the Supplement). Women presented with their first CRC at later ages than men (median difference, 3.3 years; 95% CI, 1.2-5.4 years; *P* = .002). Individuals with *MSH6* mutations presented at later ages than those with mutations in other genes: *MLH1* median difference, 9 years (95% CI, 5-14 years); *MSH2* median difference, 8 years (95% CI, 3-12 years); and *PMS2*, median difference, 6 years (95% CI, −3 to 13 years; (*P* < .001) (Figure). Furthermore, there was a trend for individuals with truncating *MLH1* mutations presenting with CRC at later ages than those with nontruncating mutations, although the difference was not statistically significant. In women, CRC was the sentinel cancer, with CRC being diagnosed before EC (median difference, 2.6 years; 95% CI, 0.2-4.8 years; *P* = .006).

### Stratified Cancer Surveillance by Mutated Gene

The number of biennial colonoscopies and annual gynecology reviews to identify cancer in individuals with LS aged 25 to 39 years is detailed in the Table. If a rate threshold of 0.5% cancers per screen is justifiable, colonoscopies starting at age 25 years for those with *MLH1* and *MSH2* mutations and at age 30 years for those with *MSH6* and *PMS2* are appropriate. For that same rate, gynecological surveillance is appropriate from age 30 years for those with *MSH2* mutations, from age 35 years for those with nontruncating *MLH1* mutations, and from age 40 years for those with *MSH6* and truncating *MLH1* mutations. Women with heterozygous *PMS2* mutations do not warrant gynecological surveillance because their absolute risk of gynecological cancer is very low.^6^

**Table.** Cancer Incidence per Mutated Gene and the Number of Colonoscopies and Gynecological Reviews That Would Be Performed if Surveillance Started at the Noted Patient Ages

Age at Surveillance Start	Mutated Gene			
	*MLH1*	*MSH2*	*MSH6*	*PMS2*
**CRC Surveillance** ^**a**^				
From age 25 y				
Colonoscopies to age 40 y, No.	2691	3181	875	294
CRCs found, ages 25-39 y, No.	81	74	7	3
Colonoscopies per CRC, %^b^	3.0	2.3	0.8	1.0
From age 30 y				
Colonoscopies to age 40 y, No.	1740	2086	579	192
CRCs found, ages 30-39 y, No.	70	66	7	2
Colonoscopies per CRC, %	4.0	3.2	1.2	1.0
From age 25 to age 30 y^c^				
Colonoscopies to age 30 y, No.	951	1095	296	102
CRCs found, ages 25-29 y, No.	11	8	0	1
Colonoscopies per CRC, %	1.2	0.7	0	1.0
**Gynecological Surveillance** ^**a**^				
From age 25 y				
Annual reviews to age 40 y, No.	2620	3616	923	293
ECs or OCs found, ages 25-39 y, No.	7 (1 OC)	18 (7 OCs)	0	0
Annual EC or OC rate, %	0.3	0.5	0	0
From age 30 y				
Annual reviews to age 40 y, No.	1688	2285	603	183
ECs or OCs found, ages 30-39 y, No.	7 (1 OC)	15 (4 OCs)	0	0
Annual EC or OC rate, %	0.4	0.7	0	0
From age 35 y				
Annual reviews to age 40 y, No.	816^d^	1108	294	90
ECs or OCs found, ages 35-39 y, No.	5	8	0	0
Annual EC or OC rate, %	0.6	1.4	0	0
From age 25 to age 30 y				
Annual reviews to age 30 y, No.	749	908	318	101
ECs or OCs found, ages 25-29 y, No.	0	3 (3 OCs)	0	0
Annual EC of OC rate, %	0	0.3	0	0

Abbreviations: CRC, colorectal cancer; EC, endometrial cancer; OC, ovarian cancer.

^a^ Number of screens assumes 2-yearly colonoscopy and annual gynecology reviews in the intervals described for all patients censored at current age, age at death, or relevant cancer (EC, OC, or CRC).

^b^ The rates of CRC and EC or OC per screen are given for each age range.

^c^ If CRC screening started aged 30 years, only 1 *PMS2* or *MSH6* mutation–associated CRC would have been missed for 398 screens, whereas for *MLH1* or *MSH2* mutations, 19 associated CRCs would have been missed for 2046 screens.

^d^ For truncating *MLH1* mutations, if screening started aged 40 years, only 1 EC would have been missed for 340 annual reviews, a rate of 0.29% between ages 35 and 39 years, whereas for nontruncating *MLH1* mutation carriers, 4 ECs would have been missed for 476 screens, a rate of 0.84%.

## Discussion

In this study, *MSH6* mutation carriers presented later with CRC and EC than those with *MLH1* or *MSH2* mutations, consistent with previous studies.^6^ Furthermore, women with truncating *MLH1* mutations presented with EC on average 6 years later than those with nontruncating mutations, which to our knowledge has not been reported before.

Among the study patients, CRC had an earlier age at onset than EC. This result is from our research group’s established surveillance program for CRC enabling earlier detection and thus younger ages of diagnosis for screened individuals.^8^ When CRC is the presenting cancer in known mutation carriers, the potential benefits of synchronous risk-reducing hysterectomy and bilateral salpingo-oophorectomy should be discussed with women.

A strength of the present study is the large LS database. Its origin as a clinical database necessitates its accuracy and prospective ongoing maintenance by a dedicated data manager. The center has extensive experience in the clinical application of DNA sequencing.^9^ While missing data could have biased the results, only 4% of data sets were incomplete.

It is interesting to speculate why patients with truncating mutations developed cancer at a later age. In ataxia-telangiectasia, missense *ATM* mutations lead to a nearly functional protein molecularly similar to the ATM protein that competes with the wild type and creates premature genomic instability and earlier disease onset. Conversely, the truncating *ATM* mutation protein is nonfunctional, and therefore, genomic stability is preserved while the wild type remains. It is not until the second knockdown mutation occurs that ATM dysfunction ensues. This is called the “dominant negative” phenomenon^10^ and could explain the later age at EC onset seen in carriers of the truncating *MLH1* mutation.

Møller et al^6^ studied the impact of mutated genes on age at cancer onset and recommended surveillance from age 25 years in carriers of *MLH1* or *MSH2* mutations and from age 40 years in those with *MSH6* or *PMS2* mutations. Some national expert guidelines corroborate these recommendations,^11^ but others endorse a standardized approach,^12^ which is simplistic and easy to implement but crude. Targeted surveillance has a de facto benefit in both reducing health care costs and decreasing patient distress.

### Limitations

The data in the present study originate from a defined geographical area in the northwest of England, and potential local population factors may limit the generalizability of the conclusions. Rare genetic conditions like LS benefit from collaborative multicenter investigation. The cumulative incidence reported should not be interpreted as the risk for mutation carriers because those diagnosed with cancer are more likely to be tested. We did not make corrections for index testing nor for the proportion of untested relatives who would be carriers.

## Conclusions

To our knowledge, this is the first study that promotes an extra tier of risk stratification according to mutation type, and not just mutated gene. Cancer surveillance could be started later for individuals with *MSH6* mutations, and surveillance for EC could be started later in those with truncating *MLH1* mutations.

## Notes

Supplement. **eTable.** Distribution of gene mutations and type of mutation, presenting age at time of cancer diagnosis, in CRC, EC and OC in proven Lynch syndrome individuals
