# Supplementary material for: Association of Mismatch Repair Mutation With Age at Cancer Onset in Lynch Syndrome: Implications for Stratified Surveillance Strategies
Source: JAMA Oncol. 2017 Aug 3;3(12):1702–6. doi: 10.1001/jamaoncol.2017.0619 (PMC5824283; doi:10.1001/jamaoncol.2017.0619)
Supplement: Supplement. — eTable. Distribution of gene mutations and type of mutation, presenting age at time of cancer diagnosis, in CRC, EC and OC in proven Lynch syndrome individuals [file jamaoncol-3-1702-s001.pdf]

Supplementary Online Content

Ryan NAJ, Morris J, Green K, et al. Association of Mismatch Repair Mutation With Age of Cancer Onset in Lynch Syndrome: Implications for Stratified Surveillance Strategies. *JAMA Oncology*. Published online August 3, 2017. doi:10.1001/jamaoncol.2017.0619

eTable. Distribution of gene mutations and type of mutation, presenting age at time of cancer diagnosis, in CRC, EC and OC in proven Lynch syndrome individuals

This supplementary material has been provided by the authors to give readers additional information about their work.

© 2017 Ryan NAJ et al. *JAMA Oncology*.

eTable. Distribution of gene mutations and type of mutation, presenting age at time of cancer diagnosis, in CRC, EC and OC in proven Lynch syndrome individuals.

**Colorectal Cancer**						
	MLH1		***MSH2***		***MSH6***	
	**Number (% of total)**	**Median age (range)**	**Number (% of total)**	**Median age (range)**	**Number (% of total)**	**Median age (range)**
**Total**	249	43 (17-93)	239	44 (18-88)	43	51 (31-73)
**Truncating**	103 (41)	44 (17-93)	109 (46)	44 (21-88)	32 (74)	53 (31-73)
**Large re-arrangement**	32 (13)	39 (21-67)	67 (28)	43 (18-77)	2 (5)	62 (61-63)
**Splice site**	94 (38)	43 (21-67)	45 (19)	46 (21-73)	3 (7)	49 (39-66)
**Other/Not known**	20 (8)	42 (28-56)	18 (7)	50 (29-87)	6 (14)	49 (35-63)
**Endometrial Cancer**						
	MLH1		***MSH2***		***MSH6***	
	**Number (% of total)**	**Median age (range)**	**Number (% of total)**	**Median age (range)**	**Number (% of total)**	**Median age (range)**
**Total**	53	49 (17-71)	83	47 (32-72)	26	53 (42-66)
**Truncating**	15 (28)	51 (34-59)	43 (52)	46 (33-65)	23 (89)	53 (42-65)
**Large re-arrangement**	9 (17)	46 (38-58)	21 (25)	51 (32-72)	0	
**Splice site**	16 (30)	48 (39-65)	17 (21)	50 (40-66)	0	
**Other/Not known**	13 (24)	48 (46-54)	2 (2)	63 (55-72)	3 (12)	52 (51-53)
**Ovarian Cancer**						
	MLH1		***MSH2***		***MSH6***	
	**Number (% of total)**	**Median age (range)**	**Number (% of total)**	**Median age (range)**	**Number (% of total)**	**Median age (range)**
**Total**	15	47 (31-69)	29	43 (24-71)	5	48 (46-50)
**Truncating**	4 (27)	51 (45-69)	14 (45)	45 (33-71)	3 (50)	50 (46-67)
**Large re-arrangement**	3(20)	46 (38-60)	5 (23)	48 (24-51)	0	
**Splice site**	6 (40)	46 (31-56)	8 (26)	34 (26-55)	1 (17)	47
**Other/Not known**	2 (13)	42 (41-43)	2 (7)	33 (25-42)	1 (17)	50

Footnote: Data from *PMS2* mutation carriers are excluded due to small numbers

© 2017 Ryan NAJ et al. *JAMA Oncology*.
